# Exploring in-hospital clinical outcomes among acute myocardial infarction patients with prior COVID-19 history

**DOI:** 10.34172/jcvtr.33107

**Published:** 2024-12-23

**Authors:** Kamal Sharma, Iva Patel, Rujuta Parikh, Maulik Kalyani, Khamir Banker, Dixit Dhorajiya, Apoorva M

**Affiliations:** ^1^Department of Cardiology, U.N.Mehta Institute of Cardiology and Research Centre (UNMICRC), Civil Hospital Campus, Asarwa, Ahmedabad-380016, Gujarat, India; ^2^Research Department, U.N.Mehta Institute of Cardiology and Research Centre (UNMICRC), Civil Hospital Campus, Asarwa, Ahmedabad-380016, Gujarat, India

**Keywords:** Severe COVID-19, SARS-CoV-2, Risk factors, Long COVID, MACE, AMI

## Abstract

**Introduction:**

Limited real-world data exist regarding cardiovascular outcomes in post-COVID-19 individuals following discharge, particularly within the Asian Indian population. This study aims to explore the association between prior COVID-19 history and in-hospital outcomes in acute myocardial infarction patients.

**Methods:**

Hospital database was searched for the patients who were diagnosed with Acute myocardial infarction (AMI) and were grouped according to absence (Group-A) or presence (Group-B) of history of severe COVID-19 hospitalization at least 3 months prior to the index event of AMI. Study primary endpoint was defined as major adverse cardiovascular events (MACE) comprising of Re-AMI, stroke, death (3P) and acute decompensated heart failure (4P), which were analyzed between these 2 study groups.

**Results:**

Of 10,581 consecutive patients of AMI, 5.33% (n=564/10,581) patients had prior history of severe SARS-CoV-2 hospitalization beyond 3 months of index AMI. Past severe Covid-19 patients presenting with AMI were more likely to be younger (59.12+11.23 years vs. 52.01+10.05 years) and younger than 40 years of age. Patients in Group B demonstrated a notably higher prevalence of diabetes, hypertension, higher Killip class, and lower presenting LVEF compared to Group A. In-hospital cardiac arrest, stroke, heart failure and all-cause death were significantly higher in Group B patients. Higher unadjusted odds ratio for in hospital death OR=5.78 (2.56-10.23), 3-P MACE OR=2.33 (1.23-8.65) and 4-P MACE OR=2.58 (1.36-5.43) were found in patients with prior history of COVID-19. After adjusting for comorbidities, the ratio for in-hospital MACE was found to be non-significant.

**Conclusion:**

Conventional risk factors and presence of comorbidities in individuals with prior history of COVID-19 hospitalization increased the risk of both 3P and 4P MACE during AMI.

## Introduction

 The coronavirus disease-2019 has infected more than 774 million of population, 7.02 million of death globally, and 45 millions of population infected in India till 28^th^ January 2024 according to WHO.^1^ Severe acute respiratory syndrome coronavirus -2 (SARS-CoV-2) causes corona virus disease (COVID-19), which leads to long term cardiovascular system, pulmonary system and multiorgan sequelae. The pathophysiology behind these sequelae are multifactorial and not well defined in details. Approximately 87.4% patients reported persistent of at least 1 symptom typically fatigue and dyspnea and 20% of patients experience chest pain as symptom after COVID-19 recovery.^2-3^

 Multiple mechanisms have been reported for cardiac damage in patients with COVID-19. One such mechanism involves SARS-CoV-2 has been found to affect via the angiotensin converting enzyme (ACE2) receptor, which mainly expresses in the heart, lungs, endothelium and kidney leading to myocardial damage in patients with COVID19 and counteracts the negative role of renin-angiotensin II with excessive activation of the RAS which causes down regulation and imbalance between RAS and ACE2 and contributes to development of hypertension, chronic heart failure, atherosclerosis and multiorgan injury. ^4-6^

 Another mechanism which causes the cardiac damages such as cytokine releases during ongoing severe inflammation may cause direct myocardial cell injury, plaque rupture and coronary thrombosis identified as a possible cause of ACS with SARS-CoV-2 infection with potential medications prescribed during the SARC-CoV-2 infection such as corticosteroids, antiviral medication and immunological drugs may have cardio-toxic side effects.^4^

 Short and long term cardiovascular sequel have been reported by a few studies in COVID-19 survivors. Persistent symptoms beyond 3 months of COVID infection has been defined as “Long COVID”.^7^ In patients with Long COVID, cardiovascular disease has been reported to be associated with higher major adverse cardiac events (MACE). The long term cardiac sequelae of COVID-19 may also be reliant on the severity of SAR-CoV-2 infection in initial stage. Myocardial damage can be caused either due to severity of the disease during the index infection or even may be a prognostic marker of future events in these patients who presented with severe COVID-19 infection in past. It is postulated that myocardial injury due to SAR-CoV-2 infection might be an important cause of post COVID-19 cardiovascular diseases or adverse events in affected patients.^8^ Other studies have reported a significant association of SARC-CoV-2 infection and readmission following COVID-19 for cardiovascular causes.

 The current study aimed to investigate the association between severe SAR-CoV-2 infection and the intermediate to long-term occurrence of acute myocardial infarction (AMI), as well as in-hospital events characterized by 3-point (3P) and 4-point (4P) major adverse cardiovascular events (MACE).

## Materials and Methods

 A total of 10 581 patients diagnosed with AMI from July-2020 to December-2021 were enrolled in the study at the largest tertiary cardiac care center of the country. Patients were grouped according to presence or absence of history of “Severe COVID-19” as per WHO definition, at least 3 months prior to AMI. The study was approved by institute’s ethics committee (IEC: UNMICRC/Allied/2021/20).

###  Study population 

**Figure 1:**
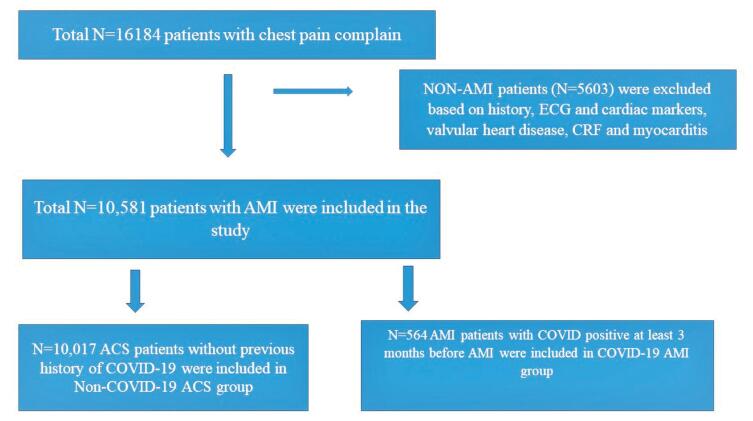
The flow chart of the study

 Patients data were retrieved from institutes electronic medical record system from July 2020 to December-2021. All patient who fulfilled study criteria were screened for the present study. Patients were evaluated for COVID-19 diagnosis and severity according to World Health Organizing guidelines. ^9^ The flow chart of the study is shown in figure 1 Demographic information included age and gender, clinical information for presence of risk factors, comorbidities and in hospital major adverse cardiac events (MACE) data were recorded. Patients with history of COVID-19 within last 3 months prior to AMI were excluded from the study. COVID-19 positive was defined by patients with presence of a positive for SARS-CoV-2 by reverse-transcriptase PCR at least before 3 months of MI while severe COVID -19 was defined as per WHO criteria as patients who were hospitalized for the same with need for oxygen or assisted ventilation and control group was constituted by patients who did not have history of COVID-19. ^10^ MACE was assessed by 3-point MACE and 4- point MACE. 3-point MACE rate was defined as combined endpoint of all-cause death, acute myocardial infarction (AMI) and stroke. 4-point MACE was defined by composite of all cause death, AMI, stroke and decompensated heart failure. Tobacco consumption was defined as having current addiction of any form of smoking or chewing or sniffing of tobacco. The AMI was defined by Forth universal definition and included ST-elevation myocardial infarction and non-ST elevation myocardial infarction, but not unstable angina. ^11^

###  Statistical analysis

 The statistical analysis was carried out using IBM SPSS (Statistical Package for Social Sciences) statistical version 26. The Chi-Square test was applied to nominal and ordinal fields yielding one-sample test to compute chi-square statistics based on the differences between the observed and expected frequencies of categories of a field. The Mean was compared in with respect to the Independent t-test (for two groups). All the descriptive statistics were presented in the form of mean ± standard deviation and percentages. Adjusted and unadjusted logistics regression was used to find association between presence of COVID-19 history and other risk factors with in hospital MACE. A nominal Statistical significance was indicated by two tailed P-value ≤ 0.05 for all analyses carried out in this investigation. The power of the study was calculated as 100% for 3-point MACE with alpha error of 0.05 for the current sample size. Propensity matched multivariate analysis was done to evaluate individual parameters where applicable.

## Results

Table 1 presents the baseline characteristics of the entire study population. AMI Patients with past history of COVID were more likely to be younger patients, diabetic, hypertensive, presenting with higher Killip class and lower LVEF compared to their COVID negative AMI cohort. Other conventional cardiovascular risk factors were found similar between both the groups.

**Table 1:** Baseline characteristics of the population

**Variables **	**Entire population (N=10,581)**	**COVID Negative-AMI (N=10,017)**	**COVID positive-AMI (N=564)**	***P*** ** value **
Female	2274 (21.49%)	2164(21.6%)	110 (19.5%)	0.291
Male	8307 (78.51%)	7853 (78.39%)	454 (80.5%)	
Age	55.57 ± 10.65	59.12 ± 11.23	52.01 ± 10.05	< 0.0001
< 40 age	1590 (15.03%)	1293 (12.9%)	297(52.7%)	< 0.0001
> 40 age	8991 (84.96%)	8724 (87.1%)	267 (47.3%)	
STEMI	6856 (64.79%)	6503 (64.92%)	353 (62.7%)	0.2791
NSTEMI	3725 (35.20%)	3514 (35.08%)	211 (37.5%)	
Comorbidities				
Diabetes	3413 (32.26%)	3201 (31.95%)	212 (37.76%)	0.01
Hypertension	2649 (25.06%)	2464 (24.6%)	185 (32.9%)	< 0.0001
Tobacco Addiction	914 (8.64%)	851 (8.5%)	63 (11.17%)	0.2428
CKD	70 (0.66%)	61 (0.6%)	09(1.59%)	0.273
Significant MR	265 (2.50%)	241 (2.40%)	15 (2.65%)	0.8098
Prior MI history	1178 (11.13%)	1134 (11.33%)	44 (7.78%)	0.3173
Previous PCI	268 (2.53%)	259 (2.58%)	9 (1.59%)	0.1875
Previous CABG	201 (1.90%)	191 (1.91%)	10 (1.77%)	0.9459
Killip class				
Killip class (mean ± SD)	1.44 ± 0.90	1.43 ± 0.88	1.74 ± 1.11	< 0.0001
II	111 (1.05%)	81 (0.81%)	30 (5.32%)	< 0.0001
III	49 (0.46%)	34 (0.34%)	15 (2.66%)	< 0.0001
IV	62 (0.58%)	53 (0.53%)	09 (1.59%)	0.002
Left ventricular ejection fraction				
LVEF (mean ± SD)	38.18 ± 11.05	39.15 ± 10.15	37.06 ± 11.96	< 0.0001
< 30	1110 (10.49%)	1025 (10.23%)	85 (15.07%)	0.0003
30-45	3106 (29.35%)	2905 (29%)	201 (35.64%)	0.001
> 45	6365 (60.15%)	6087 (60.77%)	278 (49.29%	< 0.0001

(CKD; chronic kidney disease, COPD; chronic obstructive pulmonary disease, Significant MR = Moderate to severe MR, PCI; Percutaneous coronary intervention, CABG; coronary artery bypass graft, LVEF; Left ventricular ejection fraction)

 AMI patients with a previous history of COVID-19 experienced a significantly increased incidence of cardiovascular complications including cardiac arrest, stroke, heart failure, and mortality. (Table 2)

**Table 2:** Comparison of major adverse cardiac events in both groups

**Variables **	**Entire population (N=10581)**	**COVID Negative-AMI (N=10017)**	**COVID positive-AMI (N=564)**	***P*** ** value**
Bleeding	134 (1.27%)	125 (1.24%)	09 (1.59%)	0.5993
Cardiac arrest	11 (0.10%)	08 (0.08%)	03 (0.53%)	0.01
Cardiogenic shock	60 (0.57%)	58 (0.58%)	02 (0.35%)	0.6874
Ventricular tachycardia	73 (0.69%)	72 (0.72%)	01 (0.18%)	0.2214
AV block	48 (0.45%)	46 (0.46%)	02 (0.35%)	0.9699
Stroke	13 (0.12%)	07 (0.07%)	06 (1.06%)	< 0.0001
Re-MI	07 (0.07%)	06 (0.06%)	01 (0.18%)	0.8309
Heart failure	06 (0.06%)	02 (0.02%)	04 (0.71%)	< 0.0001
Death	48 (0.45%)	21 (0.21%)	27 (4.79%)	< 0.0001
3-P MACE (Stroke, Re-MI, Death)	68 (0.64%)	34 (0.34%)	34 (6.03%)	< 0.0001
4-P MACE (Stroke, Re-MI, Death, heart failure)	69 (0.65%)	36 (0.36%)	38 (6.74%)	< 0.0001

**Figure 2:**
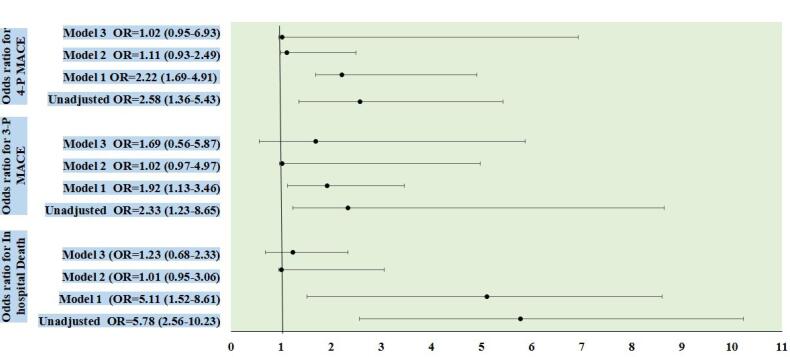
The forest plot for odds of COVID-19 history

Table 3 presents the unadjusted and adjusted odds ratio for in-hospital major adverse clinical cardiac events between the two study groups. Unadjusted odds ratio was found significantly higher for in-hospital death, 3-P MACE and 4-P MACE in AMI pateints with past history of COVID. However, after adjustment for diabetes, hypertension, smoking previous history of MI, Killip class, LVEF and moderate to severe MR; the odds of COVID-19 history for major adverse cardiac events was found to be insignificant. Figure 2 shows the forest plot for odds of COVID-19 history.

**Table 3:** Odds ratio of COVID-19 AMI for in hospital major clinical cardiac events (MACE) among study population.

	**COVID AMI** **N=564**	**Non-COVID AMI** **N=10,017**	***P*** ** value **
Odds ratio for In hospital Death			
Unadjusted OR (95% CI)	5.78 (2.56-10.23)	1	< 0.0001
Model 1 OR (95% CI)	5.11 (1.02-6.61)	1	0.05
Model 2 OR (95% CI)	1.0(0.95-3.06)	1	0.07
Model 3 OR (95% CI)	1.23 (0.68-2.33)	1	0.078
Odds ratio for 3-P MACE			
Unadjusted OR (95% CI)	2.33 (1.23-8.65)	1	0.003
Model 1 OR (95% CI)	1.92 (1.13-3.46)	1	0.005
Model 2 OR (95% CI)	1.02 (0.97-4.97)	1	0.06
Model 3 OR (95% CI)	1.69 (0.56-5.87))	1	0.695
Odds ratio for 4-P MACE			
Unadjusted OR (95%) CI	2.58 (1.36-5.43	1	0.0001
Model 1 OR (95% CI)	2.22 (1.69-4.91)	1	0.04
Model 2 OR (95% CI)	1.11 (0.99-2.49)	1	0.06
Model 3 OR (95% CI)	1.02 (0.95-6.93)	1	0.09

(Model 1 adjusted for age, Model 2 adjusted for variables included model 1 plus diabetes, hypertension, smoking, Previous history of MI, Model 3 adjusted for variables included model 1 and killip class, LVEF, Mod to Severe MR)

## Discussion

 This is one of the largest case-control Indian study evaluating the association of long COVID-19 and AMI in intermediate duration after at least 3 months of index COVID-19 hospitalization and cardiovascular outcome. We analyzed a total of 10,581 AMI patients’ datasets with and without history of COVID-19 and found 564 (5.33%) patients had severe COVID-19 at least 3 months prior to index AMI (Long COVID). Patients of AMI with prior COVID-19 were more likely to be diabetic, hypertensive, higher Killip class and lower LVEF as compared to patients without COVID-19 AMI cohort. In our outcome analysis, we observed higher odds of mortality and major adverse cardiovascular events (MACE) among patients with a history of COVID-19 driven primarily by higher stroke rates and it may be attributed to higher prevalence of diabetes, hypertension, elevated Killip class, and lower left ventricular ejection fraction (LVEF) in these individuals.

 In one of the systemic meta-analysis, higher odds of cardiovascular manifestations in post-COVID-19 cases was reported. ^12^ Previous studies have highlighted the evidence of cardiac involvement and cardiovascular complications post COVID-19 recovery. ^12-15^ Some of the previous studies have also shown that possible underlying pathophysiology responsible for the cardiac involvement. ^12,7,16-17^

 The evidence that people with COVID-19 beyond the first 30 days of infection, exhibit increased Cardiac risk and 12-month burdens of incident cardiovascular outcomes has been documented in previous studies as well. ^7^ The current findings are consistent with other previously published data on COVID-19 and cardiovascular disease related mortality. In the study by Emma et al^16^ which reported acute cardiovascular complications after COVID-19 in patients with pre exciting diabetes and CVD where they concluded that patients without any prior CV risk factor, who suffered from COVID-19, did not appear to have a long-term increase in incidence of cardiovascular diseases. However, our study found higher risk of 3-P MACE and 4-P MACE in unadjusted regression analysis but after adjustment for comorbidities and propensity matching, the CV risk found was not significant as shown in table 3 for 3P and 4P MACE.

 In a previous large study, higher mortality and cardiac arrest were reported compared to patients without COVID-19 syndrome.^18^ In another national wide registry analysis on consecutive STEMI. showed concurrent infection of SARS-COV-2 revealed a significantly higher in-hospital mortality, stent thrombosis and cardiogenic shock after PCI in patients with STEMI and COVID-19 in comparison with contemporaneous non-COVID-19 STEMI patients.^19^ Concomitant COVID-19 infection in patients with STEMI were associated with two fold higher in hospital mortality and increased mortality rates (52.56%) among patients with cardiogenic shock related to STEMI compared with patients without COVID -19.^20^

 Our study found that a significantly higher in-hospital all-cause mortality rate of 4.79% was associated with a history of prior COVID-19 compared to patients without COVID-19. On regression analysis, we found higher traditional CV risk factors along with history of Covid-19 hospitalization with higher odds for in hospital death and major adverse cardiac events. However, the odds ratio was found insignificant when risk factors and comorbidities were adjusted for MACE rates. This evidence indicates that major cardiovascular events are not solely attributed to COVID-19 history; rather, the higher prevalence of traditional CV comorbidities in patients exacerbates the likelihood of in-hospital cardiovascular events.

 Though earlier studies have reported significant myocardial injury (higher value of Troponin I and B-type natriuretic peptide) in patients admitted for COVID-19 syndrome, which shows the patient develop myocardial injury during early SARS-CoV-2 infection ^21-22^, the same has not been confirmed unequivocally in intermediate terms. The present study results are however in concurrence with a recently study showing significantly higher risk of adverse cardiovascular events and cardiovascular death in post- COVID-19 beyond acute illness. ^23^

 A relatively paucity of literature to find any association of post COVID -19 recovery and intermediate term cardiovascular events in Indian population had promoted the current study. To the best of our knowledge, the present study is the first of its kind in Asian Indians that evaluated data on cardiovascular sequelae in post COVID-19 patients from the prospect of evaluating its impact as well as role in AMI. The outcomes recommend that the emergency clinical health workers should consider the patients history of severe COVID-19 when evaluating and managing the patient with cardiovascular diseases.

 This study is a single-center observational analysis, and it does not include long-term data.

## Conclusion

 In summary, our study underscores the significance of considering both conventional risk factors and the presence of comorbidities in individuals with a prior history of COVID-19 hospitalization. This association significantly increases the risk of major adverse cardiovascular events (MACE), including both 3P and 4P MACE, during acute myocardial infarction (AMI). Such findings emphasize the importance of comprehensive risk assessment and tailored management strategies for this vulnerable patient population to mitigate adverse outcomes and improve overall clinical care.

## Competing Interests

 None.

## Ethical Approval

 The study was approved by institute’s ethics committee (IEC: UNMICRC/Allied/2021/20) on date 16/09/2021.
